# Persistent Left Superior Vena Cava in Patients Undergoing Cardiac Device Implantation: Clinical and Long-Term Data

**DOI:** 10.4021/cr267w

**Published:** 2013-05-09

**Authors:** Dubravko Petrac, Vjekoslav Radeljic, Nikola Pavlovic, Sime Manola, Diana Delic-Brkljacic

**Affiliations:** a Department of Cardiology, Sestre Milosrdnice University Hospital Center, Zagreb, Croatia

**Keywords:** Persistent left superior vena cava, Cardiac device implantation, Long-term outcome

## Abstract

**Background:**

Persistent left superior vena cava (LSVC) is a rare congenital venous anomaly that may be found at the time of cardiac device lead insertion.

**Methods:**

In this case series, we present clinical and long-term data of five patients with LSVC who underwent pacemaker (PM) or cardioverter defibrillator (ICD) implantation during the period of 10 years.

**Results:**

Left-sided venous approach was used for device implantation in 3 patients with standard PM indications, whereas a right-sided venous approach and an epicardial approach had to be used in 2 patients who needed an ICD and biventricular PM, respectively. In post implantation period of 44 ± 29 months, one patient died due to stroke, one underwent heart transplantation, and 3 had atrial fibrillation.

**Conclusion:**

The long-term outcome of patients with persistent LSVC and implanted cardiac devices is mostly influenced by the presence of underlying heart disease.

## Introduction

Persistent LSVC is a rare congenital venous anomaly [1] that can be found at the time of cardiac device lead insertion. In most cases, persistent LSVC exists together with the right superior vena cava (RSVC) and communicates to the right atrium via coronary sinus [2]. Isolated LSVC is a rare variation, characterized by dilated coronary sinus [3]. Others rare variations include a complete unroofed coronary sinus [4], sinus ostial atresia with cardiac venous drainage into the subclavian vein [5] and an absence of any communication between LSVC and the coronary sinus, left atrium or pulmonary venous system [6]. Although the first transvenous pacemaker implantation by way of anomalous LSVC was reported as early as in 1971 [7], clinical experience with these patients, particularly in the post implantation period, is still small. In this article, we present the clinical and long-term data of 5 patients with persistent LSVC who underwent cardiac device implantation.

## Materials and Methods

**Figure 1:**
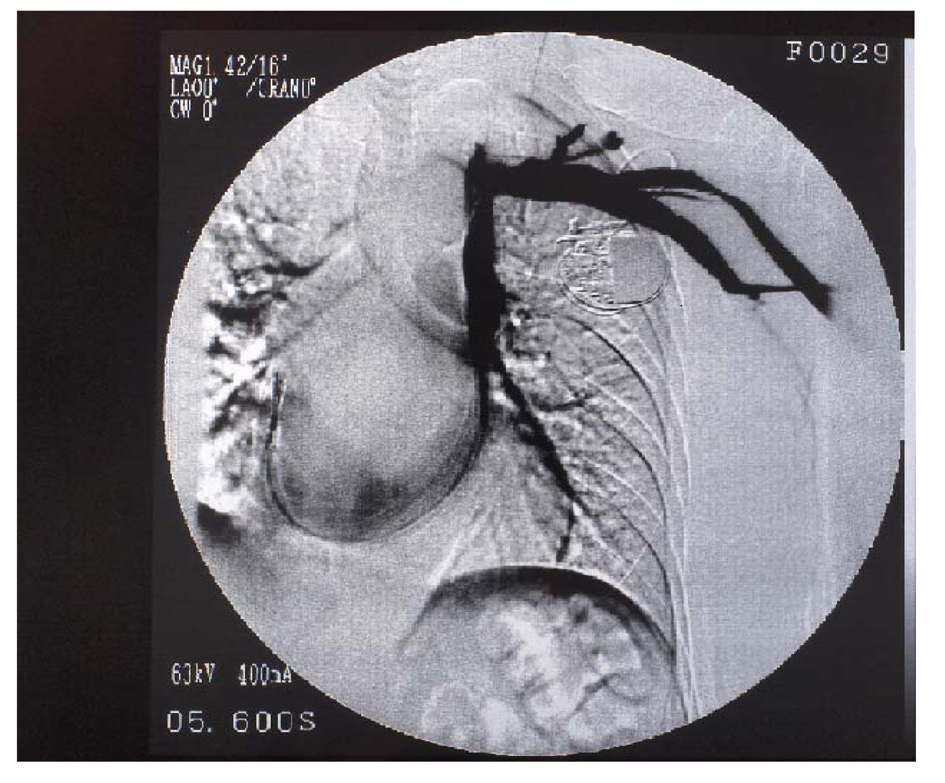
Digital substraction angiography of the upper venous system in patients with a right atrial pacing. Persistent left vena cava superior drains through the coronary sinus to the right atrium and serves as a truck for the atrial lead placement (patient 1).

Our case series consists of patients with persistent LSVC who underwent pacemaker (PM) or ccardioverter-defibrillator (ICD) implantation between August 2000 and August 2010. The persistent LSVC was recognized during the device implantation when the guide wire entered the left subclavian vein but did not cross on the right side of vertebral column before entering the coronary sinus. Diagnosis of this anomaly was confirmed later by digital subtraction angiography (DSA) (Fig. 1) or multislice computed tomography (MSCT). After device implantation, all patients with persistent LSVC were seen in our pacemaker center every six month. Each follow-up visit included clinical, electrocardiographic and implanted device evaluation.

## Results

During a 10-year period, persistent LSVC was found in five (0.35%) out of 1390 patients undergoing first PM (n = 1,296) or ICD (n = 94) implantation. Four patients had persistent LSVC and normal right superior vena cava (RSVC), whereas one patient (patient 2) had an absent RSVC. Clinical and long-term data of these patients are shown in Table 1.

**Table 1:** Clinical and Long-Term Data of Study Patients

Patient number	Age Sex	Device indication	Heart disease LVEF	Device type	Implantation approach	Follow-up (months)	Outcome
1	68 F	Sinus node dysfunction	Hypertension 60%	PM AAI	Left subclavian	124	Alive and well
2	80 M	Chronic AF and slow ventricular rate	Hypertension 45%	PM VVIR	Left subclavian	28	Chronic AF, died due to stroke
3	53 M	Unstable VT	Piror MI 35%	ICD	Right subclavian	28	Alive, paroxysmal AF
4	42 M	Sinus node dysfunction	None 65%	PM DDDR	Left subclavian	23	Alive, asymptomatic AF
5	51 F	NYHA class III/IV and LBBB	Nonischemic CM 25%	CRT-P	Epicardial	18	Alive, heart transplantation

LVEF: left ventricular ejection fraction; F: female; PM: pacemaker; AAI: atrial single-chamber pacemaker; M: male; AF: atrial fibrillation; VVIR: ventricular single-chamber rate-adaptive pacemaker; MI: myocardial infarction; VT: ventricular tachycardia; ICD: implantable cardioverter defibrillator; NYHA: New York Heart Association; CRT-P: pacemaker with cardiac resynchronzation therapy; LBBB: left bundle branch block; CM: cardiomyopathy.

**Figure 2:**
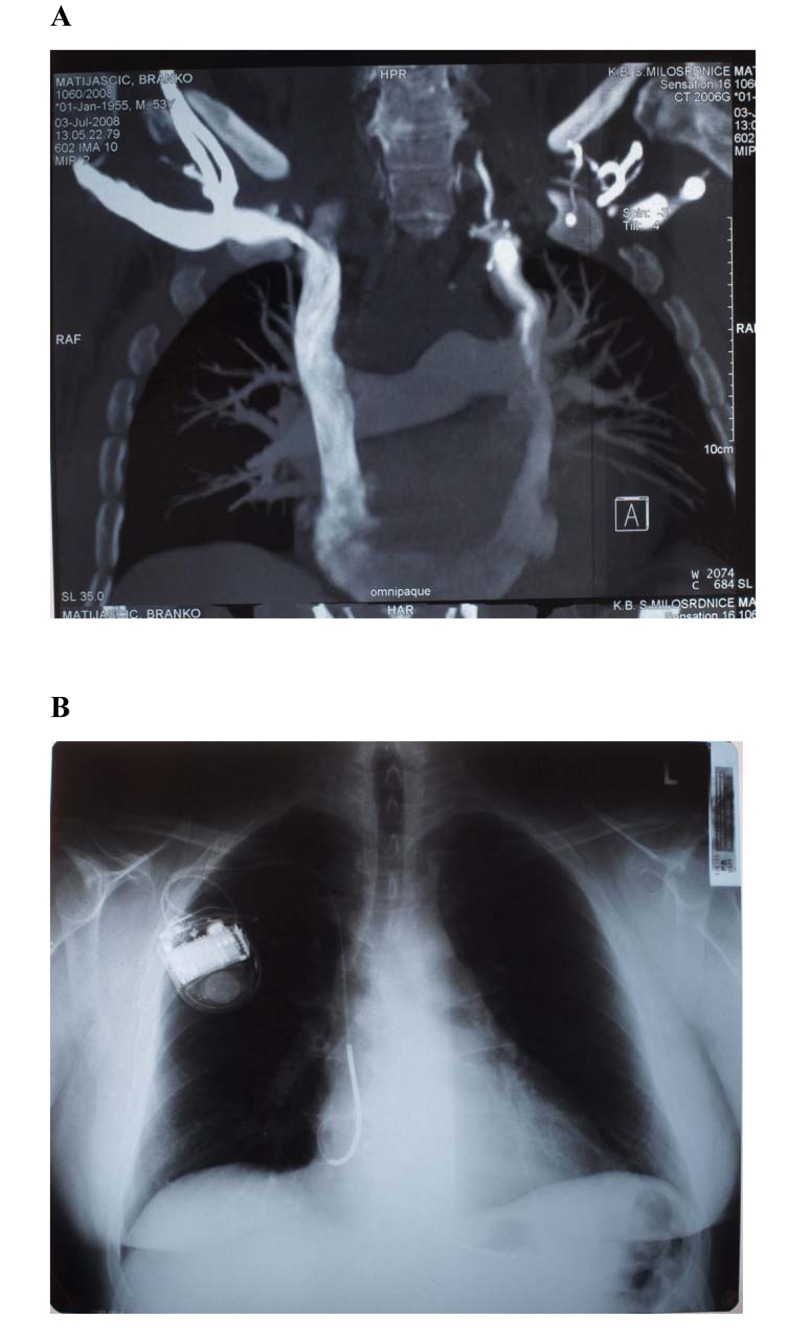
A) Multislice CT-angiography showing a persistent left superior vena cava with a presentation of right subclavian vein. B) A quadripolar dual coil active defibrillation lead was inserted in the right ventricle via right subclavian vein and connected with an ICD impalanted at right pectoral side (patient 3).

In three patients with standard pacemaker indications, atrial and/or ventricular electrodes were successfully inserted via persistent LSVC and coronary sinus using a hand-shaped stylet and an active fixation lead. In the third patient, we could not achieve a stable position of defibrillation lead in the right ventricle during the shock delivery. Since the RSVC was present on MSCT (Fig. 2A), an ICD with a double-coil defibrillation lead was implanted using the right subclavian approach (Fig. 2B). A sufficient defibrillation threshold (< 15 joules) was obtained by turning the active can off so that the vector of shock was directed between right atrium coil and right ventricular coil. In the fifed patient, the anatomy of coronary sinus was not suitable for a transvenous insertion of left ventricular pacing lead. Therefore, the epicardial approach with inferior sternotomy was used. One lead was fixed to the right atrium, one to anterior wall of the right ventricle, and one to lateral wall of the left ventricle. All leads were tunneled to the right sub-costal pocket where a device for cardiac resynchronization therapy was implanted.

After cardiac device implantation, patients were followed-up for 44 ± 29 months. Among three patients with standard PM implantation, one with chronic AF died due to stroke despite of warfarin therapy, one had asymptomatic episodes of AF recorded by implanted PM, and one underwent PM replacement at 120 months of follow-up. A patient with implanted ICD developed paroxysmal AF and had several episodes of VT, successfully terminated by ICD therapy. Because of that, amiodarone was introduced for the treatment of both arrhythmias. A patient with dilated cardiomyopathy was not good responder to cardiac resynchronization therapy, and 18 months later, she underwent heart transplantation.

## Discussion

Persistent LSVC occurs in 0.3% of individuals in the general population [8] and in 3% to 10% of patients with congenital heart disease [2]. Prevalence of this abnormality in adult PM and ICD population is low and ranges from 0.6% to 0.41% [9, 10]. In our series, the prevalence of persistent LSVC was similar to that in the previous reports, and almost the same with that in general population (0.35% versus 0.3%).

The transvenous placement of cardiac device lead via persistent LSVC can be technically difficult, and in some cases, impossible [9, 11, 12]. The major difficulty relates to the right ventricular lead implantation, as the tip of the lead is deflected away from the tricuspid annulus, or to the placing of the left ventricular pacing lead in the coronary sinus branch. Using various techniques [12-15], there was possible to insert cardiac leads via persistent LSVC to the appropriate site within the heart in about 80% of patients [9, 10, 13]. In our experience, lead placement by the left subclavian approach was successful in 3 patients with persistent LSVC, who had standard indication for cardiac pacing. In two patients, the implantation approach via persistent LSVC could not be used because of an unstable position of defibrillation lead in the right ventricle, or non suitable coronary sinus anatomy for the left ventricular pacing. Therefore, a right sided venous approach was used in a patient who needed an ICD, and an epicardial approach in a patient requiring biventricular PM. To resolve such problems, some authors used an additional subcutaneous patch for the sufficient defibrillation threshold [10, 14, 16], tunneled a right-sided defibrillation lead to the left pectoral pocket [12], or used a left lateral thoracotomy for the placing of left ventricular lead [9].

Regarding to clinical course, persistent LSVC is usually asymptomatic without additional congenital heart defects. Patients with a persistent LSVC may become symptomatic due to arrhythmias through fragmentation and stretching of the conduction tissue by dilated coronary sinus or ectopic pacemaker cells [2, 17]. The presence of sinus node dysfunction observed in our 2 patients with persistent LSVC could also reflect a mal development of the sinus node, or a lesion of its nutrient artery [18]. There is some evidence that LSVC, as a source of ectopy, can initiate AF in patients with persistent LSVC despite previous pulmonary vein isolation [17, 19]. In such patients, Hsu et al. found electrical connections between LSVC and coronary sinus and LSVC and left atrium [19]. Ablation of these connections resulted in electrical isolation with a maintenance of sinus rhythm without additional antiarrhythmic drugs.

Our follow-up data show that the long-term outcome of patients with persistent LSVC and implanted cardiac devices is mostly influenced by the presence of underlying heart disease. Accordingly, a patient with hypertension and chronic AF died due to stroke, a patient with a prior myocardial infarction continued to have VT episodes, and a patient with chronic congestive heart failure underwent heart transplantation. These findings are consistent with the earlier reports, in which two patients with ischemic dilated cardiomyopathy underwent heart transplantation [10] and another died due to progression of ischemic heart disease [20]. Development of AF in our three patients could be associated with an arrhythmogenic substrate of persistent LSVC, but in two of them we can not exclude the underlying heart disease as a cause of this arrhythmia. Although the prevalence of AF in LVSC patients is unknown because of rarity of this malformation, these patients, as other AF population, require thromboembolic risk assessment and timely initiated therapy, which should be directed either to the AF substrate and underlying heart disease.

In conclusion, persistent LSVC may severely complicate left-sided PM or ICD implantation and require alternative approaches for the lead insertion. The long-term outcome of patients with persistent LSVC and implanted cardiac devices is mostly influenced by the presence of underlying heart disease.
